# Carnosic Acid Alleviates Levodopa-Induced Dyskinesia and Cell Death in 6-Hydroxydopamine-lesioned Rats and in SH-SY5Y Cells

**DOI:** 10.3389/fphar.2021.703894

**Published:** 2021-08-09

**Authors:** Chun-Yi Lai, Chia-Yuan Lin, Chi-Rei Wu, Chon-Haw Tsai, Chia-Wen Tsai

**Affiliations:** ^1^ Department of Nutrition, China Medical University, Taichung, Taiwan; ^2^ Department of Chinese Pharmaceutical Sciences and Chinese Medicine Resources, China Medical University, Taichung, Taiwan; ^3^ Department of Neurology, China Medical University Hospital, Taichung, Taiwan; ^4^ College of Medicine, China Medical University, Taichung, Taiwan; ^5^ Graduate Institute of Acupuncture Science, College of Chinese Medicine, China Medical University, Taichung, Taiwan

**Keywords:** carnosic acid, 6-hydroxydopamine, levodopa-induced dyskinesia, DARPP-32/ΔFosB, ERK1/2-c-jun

## Abstract

The present study investigated the impact of carnosic acid (CA) from rosemary on the levodopa (_L_-dopa)-induced dyskinesia (LID) in rats treated with 6-hydroxydopamine (6-OHDA). To establish the model of LID, 6-OHDA-lesioned rats were injected intraperitoneally with 30 mg/kg _L_-dopa once a day for 36 days. Rats were daily administrated with 3 or 15 mg/kg CA by oral intubation prior to _L_-dopa injection for 4 days. Rats pretreated with CA had reduced _L_-dopa-induced abnormal involuntary movements (AIMs) and ALO scores (a sum of axial, limb, and orofacial scores). Moreover, the increases of dopamine D1-receptor, p-DARPP-32, ΔFosB, p-ERK1/2, and p-c-Jun ser63, along with the decrease in p-c-Jun ser73, induced by _L_-dopa in 6-OHDA-treated rats were significantly reversed by pretreatment with CA. In addition, we used the model of SH-SY5Y cells to further examine the neuroprotective mechanisms of CA on _L_-dopa-induced cytotoxicity. SH-SY5Y cells were treated with CA for 18 h, and then co-treated with 400 μM _L_-dopa for the indicated time points. The results showed that pretreatment of CA attenuated the cell death and nuclear condensation induced by _L_-dopa. By the immunoblots, the reduction of Bcl-2, p-c-Jun ser73, and parkin and the induction of cleaved caspase 3, cleaved Poly (ADP-ribose) polymerase, p-ERK1/2, p-c-Jun ser63, and ubiquitinated protein by _L_-dopa were improved in cells pretreated with CA. In conclusion, CA ameliorates the development of LID *via* regulating the D1R signaling and prevents _L_-dopa-induced apoptotic cell death through modulating the ERK1/2-c-Jun and inducing the parkin. This study suggested that CA can be used to alleviate the adverse effects of LID for PD patients.

## Introduction

Parkinson’s disease (PD) is a progressive neurodegenerative disease. Levodopa (_L_-dopa), the precursor of dopamine, is the primary drug used to treat PD (Cenci, 2014). However, long-term exposure to _L_-dopa causes motor complications, called levodopa-induced dyskinesia (LID) (Chaudhuri et al., 2019). LID develops in about 40–50% of patients in the 5 years after treatment and in up to 100% of patients after 10 years of treatment (Manson et al., 2012). The symptoms of LID include chorea, ballism, dystonia, and myoclonus (Calabresi et al., 2010). New adjunct therapies to delay or reduce these adverse effects are needed.

The mechanisms by which LID develops are complex. LID is associated with the hypersensitization of striatum dopamine D1-receptor (D1R) induced by impaired receptor trafficking (Feyder et al., 2011; Spigolon and Fisone, 2018), which results in the abnormal change of proteins of PKA, ΔFosB, and extracellular signal-regulated kinases1/2 (ERK1/2) (Fieblinger et al., 2014). An evidence has shown that pulsatile administration of _L_-dopa activates the proteins of DARPP-32, ERK, and ΔFosB in 6-OHDA-lesioned rats (Lebel et al., 2010). _L_-Dopa stimulates D1R coupled with G-protein α/olf to activate PKA, and then phosphorylates DARPP-32 protein at Thr34, leading to increase the ERK1/2 activity. The activation of ERK1/2 results in increased the activation of ΔFosB by cAMP response element-binding protein (CREB) (Fanni et al., 2018; Spigolon and Fisone, 2018). Striatal ΔFosB protein is correlated with the severity of LID in _L_-dopa-treated monkeys (Berton et al., 2009). ΔFosB is also found in the striatum of PD patients treated with _L_-dopa (Lindgren et al., 2011). Silencing of striatal ΔFosB-expression neurons improves the motor complication of LID in PD rat model (Engeln et al., 2016). Conversely, overexpression of ΔFosB in 6-hydroxydopamine (6-OHDA)-treated rats exacerbates the effects of LID (Cao et al., 2010).

Accumulating evidence indicates that cells cultured with _L_-dopa induced neurotoxicity *via* generating oxidative damage (Colamartino et al., 2015). It has been shown that the process of dopamine synthesis from _L_-dopa enhances the formation of reactive oxygen species (ROS) *via* regulating monoamine oxidase (Stansley and Yamamoto, 2013). This induction is enhanced for PD patients treated with _L_-dopa and leads to the apoptotic neuronal cell death (Hald and Lotharius, 2005; Dorszewska et al., 2014). Consistent with the results, the increment of cleaved-caspase 3 and -poly (ADP-ribose) polymerase (PARP) proteins is observed in PD patients treated with _L_-dopa (Hald and Lotharius, 2005). In addition to oxidative stress, study indicates that _L_-dopa-induced cell death is mediated by ERK1/2-c-Jun pathway (Park et al., 2016). The transcription factor c-Jun regulates cell survival and cell death through regulating its phosphorylation sites by ERK1/2 pathway. An observation by Park et al. suggested that the neurotoxicity caused by long-term _L_-dopa administration may involve the induction of c-Jun phosphorylation at ser63 and reduction of c-Jun phosphorylation at ser73, which acts as a pro- or anti-apoptotic factor, respectively (Park et al., 2016). An understanding of the possible of neurotoxicity by _L_-dopa in PD will help to improve the motor complication of LID.

Carnosic acid (CA), a diterpene phenolic compound from rosemary, has multiple physiological benefits, including anti-oxidant and neuroprotective (Guo et al., 2016; Lin et al., 2020). In our previous study, we reveals that the neuroprotective mechanisms of CA are related to the upregulation of anti-oxidant enzymes in PD models (Chen et al., 2012; Wu et al., 2015). CA stimulates glutathione synthesis to inhibit 6-hydroxydopamine (6-OHDA)-induced apoptosis of SH-SY5Y cells through down-regulating of c-Jun NH_2_-terminal kinase (JNK) and p38 (Chen et al., 2012). Furthermore, administration of CA with 6-OHDA-lesioned rats improves the antioxidant capacity, neurotoxicity, and motor impairment (Wu et al., 2015). Although CA is currently being investigated for therapeutic benefits in PD, the actions of CA on the development of LID have not yet been described. Therefore, in this study, we explored the roles of CA on LID in 6-OHDA-lesioned rats and further examined the cytotoxicity of _L_-dopa in SH-SY5Y cells.

## Materials and Methods

### Experimental Animals and Treatments

Eight-week-old male Wistar rats (BioLASCO Experimental Animal Center, Taipei, Taiwan) were used in this study. The protocols for animal-related experiments were approved by the Institutional Animal Care and Use Committee of China Medical University (protocol no. 2017–189). The temperature of the animal husbandry rooms was set at 23 ± 1°C with a 12-h day and night cycle. Animals had ad libitum access to a chow diet and water. After 11 days of adaptation, 6-OHDA (Sigma, St. Louis, MO, United States) injury surgery was performed as described below to induce PD. On day 17 after lesion formation, an apomorphine (0.25 mg/kg)-induced rotation experiment (exhibited >7 full turns/min) were performed to select for next experiment (Zhang et al., 2018). The experimental groups were as follows: 1) vehicle group (6-OHDA lesion, *n* = 5); 2) _L_-dopa group: 30 mg/kg _L_-dopa + 15 mg/kg benserazide (*n* = 5); 3) _L_-dopa + CA3 group: 30 mg/kg _L_-dopa + 15 mg/kg benserazide +3 mg/kg CA (*n* = 5); 4) _L_-dopa + CA15 group: 30 mg/kg _L_-dopa + 15 mg/kg benserazide +15 mg/kg CA (*n* = 5). _L_-Dopa (Sigma, St. Louis, MO, United States) and benserazide (Sigma, St. Louis, MO, United States) were injected intraperitoneally with potassium phosphate buffer once per day for 36 consecutive days. In the CA-treated group, CA (Cayman, Ann Arbor, MI, Cat No.89820) was dissolved in 0.5% sodium carboxymethyl cellulose and was administered 4 days before the _L_-dopa injection by oral intubation once per day until the rats were sacrificed.

### 6-OHDA Injury Surgery

According to a previous study in our laboratory (Wu et al., 2015), rats were anesthetized with tiletamine/zolazepam (Zoletil50^®^; Virbac Lab., Carros, France) by intramuscular injection. The rats were fixed on a stereotaxic apparatus and 2.5 μl of 6-OHDA (5 μg/μl) was injected into the right striatum (anteroposterior: +1.5 mm; lateral: −4 mm; dorsoventral: −7.2 mm) with a flow rate of 1 μl/min. The drug delivery tube was left in place for 1 min before being removed to avoid seepage of 6-OHDA. The sham operation group was injected with 0.5% ascorbic acid-saline.

### Abnormal Involuntary Movement Scores

Behavioral analysis subtypes and scoring criteria were based the study by Winkler et al. (Winkler et al., 2002). AIM scores were assessed on days 12, 26, and 33 after _L_-dopa administration. The rats were placed in a transparent cage for a 2-h photographic record and were scored for 1 min every 20 min. Each rat was assessed for four AIM subtypes: axial, limb, orofacial, and locomotor movements. A severity score ranging from 0 to 4 was assigned for each AIM subtype. The AIM scores from four subtypes were summed for each time point. The ALO score (sum of the axial, limb, and orofacial scores) is more responsive to human dyskinesia than are locomotive scores (Castello et al., 2020). Therefore, the ALO score was also analyzed in this study.

### Preparation of Animal Tissues

After the animals were sacrificed, the striatum on the right side was separated and homogenized (10% w/v) in RIPA buffer (Biokit, Taiwan) containing 1% protease inhibitor (Sigma, St. Louis, MO, United States) and 1% phosphatase inhibitor (Sigma, St. Louis, MO, United States). The supernatant was obtained after centrifugation at 15,000 rpm for 30 min at 4°C.

### Cell Culture and Sample Preparation

The SH-SY5Y cells were purchased from American Type Culture Collection (ATCC, Manassas, VA, United States). The method of cell culture was based on our previous study (Lin et al., 2016). The cells were plated on 3.5 cm cell culture dishes and incubated with Dulbecco’s modified Eagle medium (DMEM) including 10% FBS, 1% L-glutamine, 1% non-essential amino acid, 1 mM sodium pyruvate, 1.5 g/ml sodium bicarbonate, 1% penicillin-streptomycin; pH = 7.4. Cells were pretreated with 0.1% dimethylsulfoxide (DMSO) or 0.5, 1, or 3 μM CA for 18 h followed by treatment with PBS or 20, 200, or 400 μM _L_-dopa for an additional 24 or 72 h. After treatment, the RIPA buffer containing 1% protease inhibitor and 1% phosphatase inhibitor was added to each plate and the cells were collected for protein analysis. Lysates were centrifuged at 14,000 rpm for 20 min at 4°C to obtain the supernatant. The protein assay dye reagent concentrate (BIO-RAD, Hercules, CA, United States) was used to measure protein concentration.

### Western Blot Analysis

Samples containing the same protein concentrations were subjected to sodium dodecyl sulfate-polyacrylamide gel electrophoresis and then transferred to polyvinylidene fluoride membranes (Millipore, Bedford, MA, united States). The membranes were placed in 50 g/L skim milk at 4°C to block the nonspecific binding sites. The membranes were incubated with primary antibodies, including D1R (Santa Cruz Biotechnology (SCBT); sc-33660), p-DARPP-32 (GeneTex; GTX82714), ΔFosB (Cell Signaling Technology (CST); #14695), p-ERK1/2 (SCBT; sc-7383), ERK1/2 (SCBT; sc-93), p-c-Jun ser63 (SCBT; sc-822), p-c-Jun ser73 (CST; #3270), Bcl-2 (CST; #2876), caspase 3 (CST; #9662), cleaved caspase 3 (CST; #9661), PARP (CST; #9542), cleaved PARP (CST; #9541), parkin (SCBT; sc-32282), ubiquitin (Sigma-Aldrich; 05–944), β-tubulin (SCBT; sc-9104) and GAPDH (SCBT; sc-365062) at 4°C overnight and were then subsequently incubated with horseradish peroxidase-conjugated goat anti-rabbit (SCBT; sc-2004), goat anti-mouse IgG (SCBT; sc-2005), or mouse IgG kappa binding protein (m-IgGκ BP)-HRP (SCBT; sc-516102) secondary antibodies. The protein expression on the membrane was detected with an enhanced chemiluminescence reagent (Millipore, Burlington, MA, United States) and analyzed by a luminescent image analyzer (LAS-4000, FUJIFILM).

### Cell Viability Assay

Cells were washed with phosphate-buffered saline and incubated with DMEM containing 0.5 mg/ml 3-(4,5-dimethylthiazol-2-yl)-2,5-diphenyltetrazolim bromide (MTT) for 2 h at 37°C. After the medium was removed, the formazan crystal was dissolved with 1 ml of isopropanol and then centrifuged at 15,000 rpm for 5 min to get the supernatant. The supernatant was added to a 96-well plate, and absorbance was measured at 570 nm by ELISA (Bio-Rad, Japan). The value in the control cells was set as 100% viability.

### Nuclear Staining With Hoechst 33258

Cells were washed with phosphate-buffered saline and fixed with 3.7% paraformaldehyde (pH 7.4) solution for 50 min. Then, the cells were stained with Hoechst 33258 for 1 h at 25°C in the dark and morphological changes were observed by using a fluorescence microscope. Fluorescence intensity was obtained by use of Image-Pro Plus 6.0 (Media Cybernetics, Inc., Bethesda, MD, United States).

### Statistical Analysis

All *in vivo* data are presented as the mean ± SEM. The *in vitro* data are expressed as mean ± SD. Statistical analysis was performed with t-tests between two sample comparisons. The multiple comparisons were conducted with a one-way analysis of variance (ANOVA) with SAS software followed by Tukey’s post hoc test. Statistically significant differences were considered when the *p*-value was less than 0.05.

## Results

### CA Improves AIMs Induced by _L_-Dopa in Lesioned Rats

**FIGURE 1:**
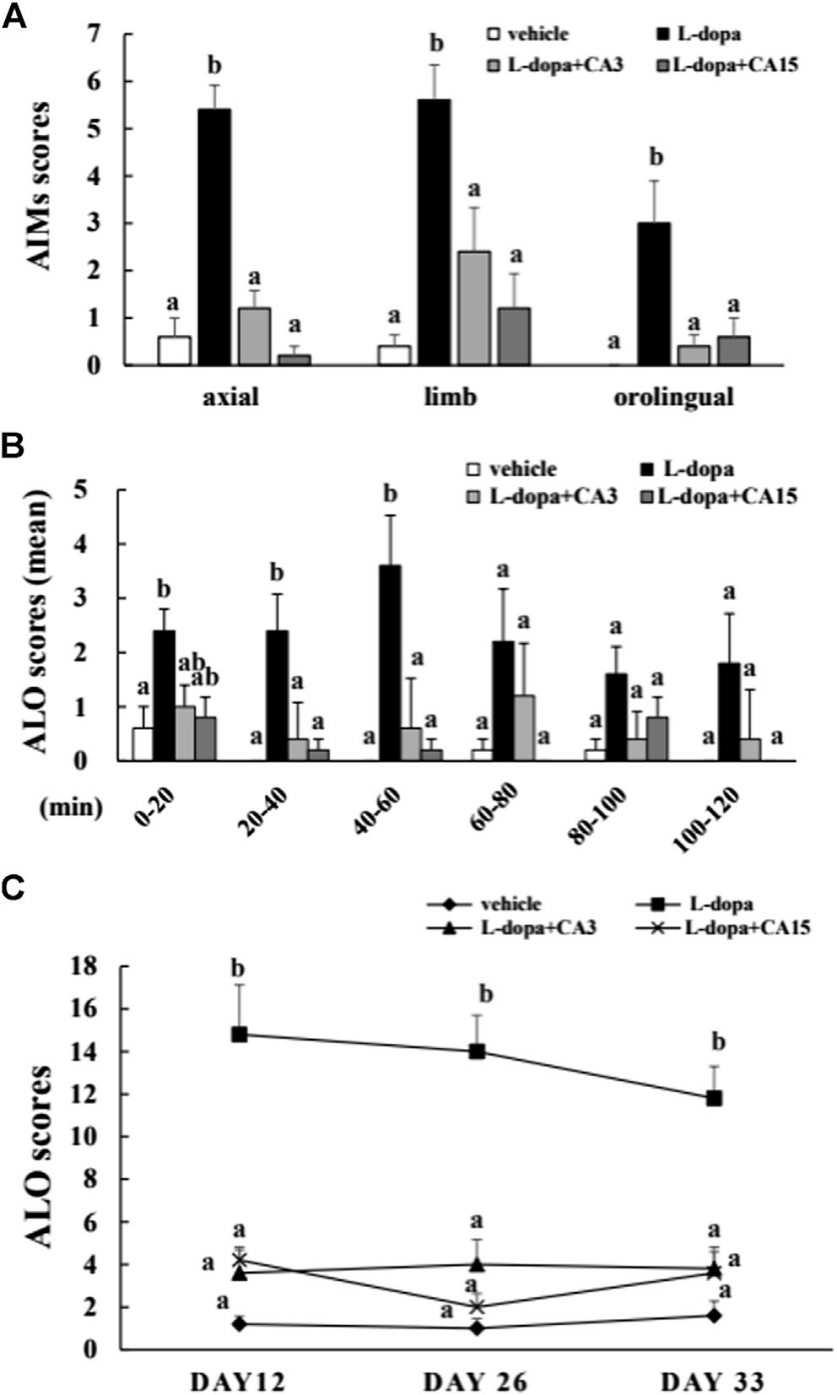
Effect of CA on _L_-dopa-induced abnormal involuntary movements (AIMs) in 6-OHDA lesioned rats. The axial, limb, and orofacial AIMs were calculated on days 12, 26, and 33 for 1 min every 20 min over 120 min. **(A)** The integrated AIM scores of axial, limb, and orofacial subtype on day 26 over 120 min. **(B)** The ALO score (the sum of axial, limb, and orofacial) on day 26 for 1 min every 20 min over 120 min. **(C)** The ALO score on days 12, 26, and 33. The results are expressed as mean ± SEM (*n* = 5). Means without a common letter differ, *p* < 0.05.

The axial, limb, and orofacial scores on day 26 in the _L_-dopa group were significantly higher than in the vehicle group (*p* < 0.05), whereas those in the groups pretreated with 3 or 15 mg/kg CA were significantly lower than in the _L_-dopa group (*p* < 0.05) (Figure 1A). We then divided the total recording time into 20-min intervals, and used a total of 6 intervals for score statistics. In this analysis, CA improved the ALO score (the sum of the axial, limb, and orofacial scores) during the 20–40 min and 40–60 min intervals (Figure 1B). In addition, the ALO scores in the _L_-dopa group were significantly higher than in the vehicle group on days 12, 26, and 33. However, the ALO scores in the group pretreated with 3 and 15 mg/kg CA were lower compared with those in the _L_-dopa group (*p* < 0.05) (Figure 1C). These results suggest that CA can improve AIMs caused by _L_-dopa.

### CA Reduces LID Marker Proteins in Lesioned Rats

**FIGURE 2:**
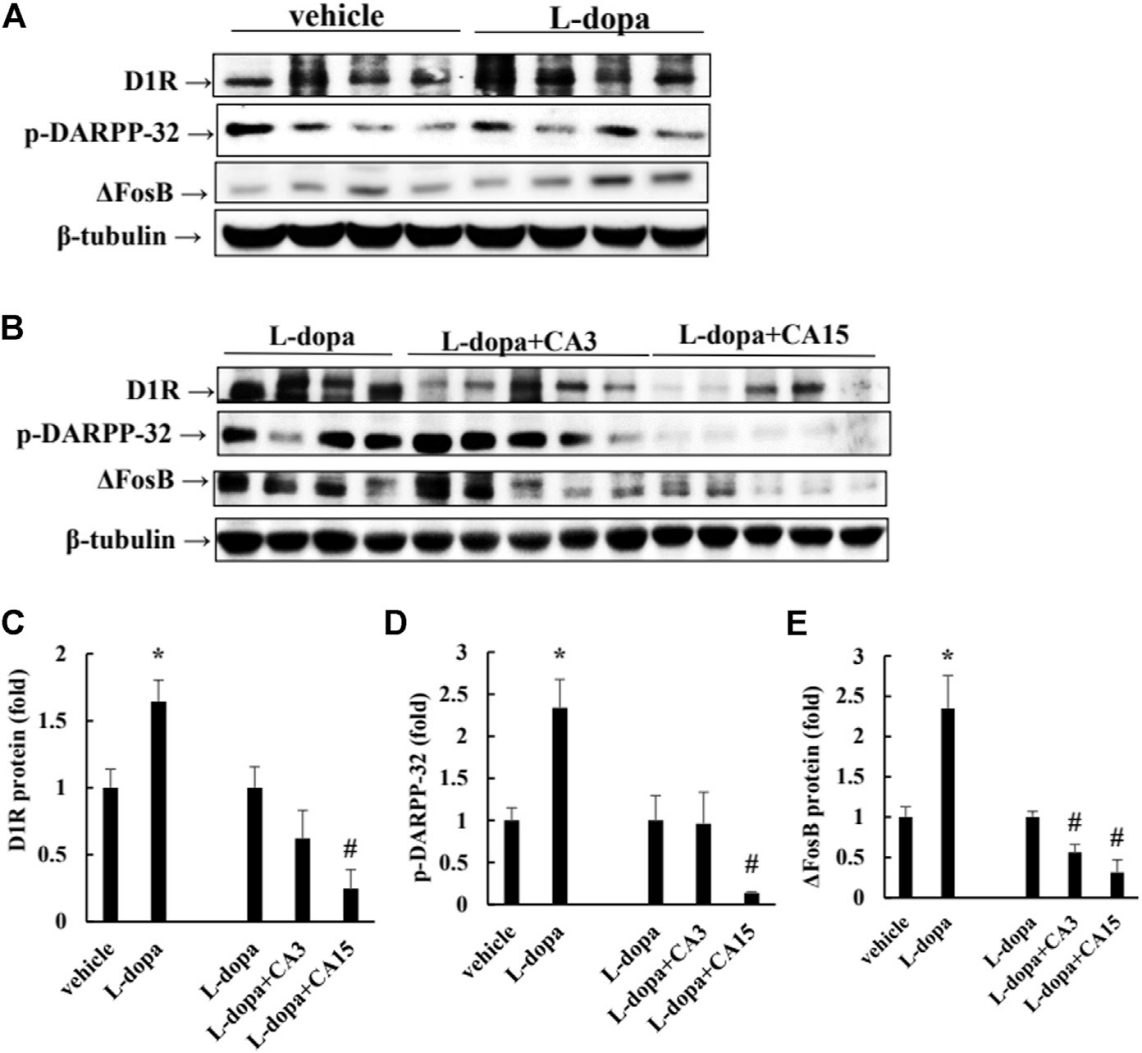
Effect of CA on _L_-dopa-induced D1R, p-DARPP-32, and ΔFosB protein in 6-OHDA-lesioned rats. **(A,B)** D1R, p-DARPP-32, and ΔFosB protein were determined by Western blotting. β-Tubulin was used as the internal control. **(C–E)** The level of the _L_-dopa group was set at 1.0. Values are mean ± SEM (*n* = 3). #*p* < 0.05 compared with _L_-dopa-treated group.

Over-activation of D1R phosphorylates DARPP-32, which ultimately leads to an increase in FosB family proteins, which causes LID (Ahn et al., 2017). Rats treated with _L_-dopa significantly increased D1R and p-DARPP-32, as well as ΔFosB protein (*p* < 0.05). Pretreatment of rats with CA at 15 mg/kg prior to 4 days of _L_-dopa treatment, significantly reduced D1R and p-DARPP-32 compared with that in the _L_-dopa group (*p* < 0.05). However, pretreatment with 3 mg/kg CA had no significant effect. ΔFosB proteins were reduced in the group pretreated with 3 and 15 mg/kg CA by 44 and 69%, respectively, compared with the _L_-dopa group (Figure 2). This result suggests that CA reduced the activation of D1R and DARPP-32, leading to improved accumulation of ΔFosB protein induced by _L_-dopa.

### Effect of CA on the Phosphorylation of ERK1/2 and c-Jun in Lesioned Rats

**FIGURE 3:**
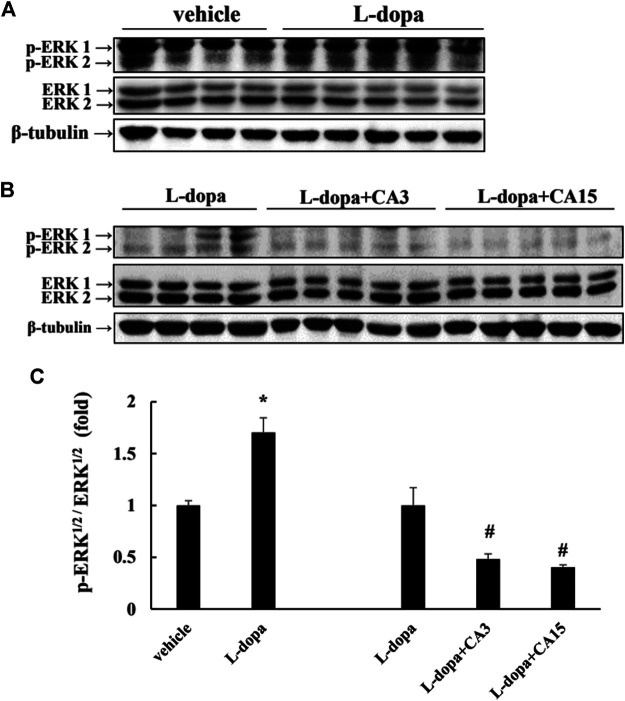
Effect of CA on _L_-dopa-induced activation of ERK1/2 and c-Jun in 6-OHDA lesioned rats. The phosphorylation of ERK1/2 **(A,B)** in striatum tissues were determined by Western blotting. β-Tubulin was used as the internal control. **(C)** The level of the _L_-dopa group was set at 1.0. Values are mean ± SEM (*n* = 3). #*p* < 0.05 compared with _L_-dopa-treated group.

**FIGURE 4:**
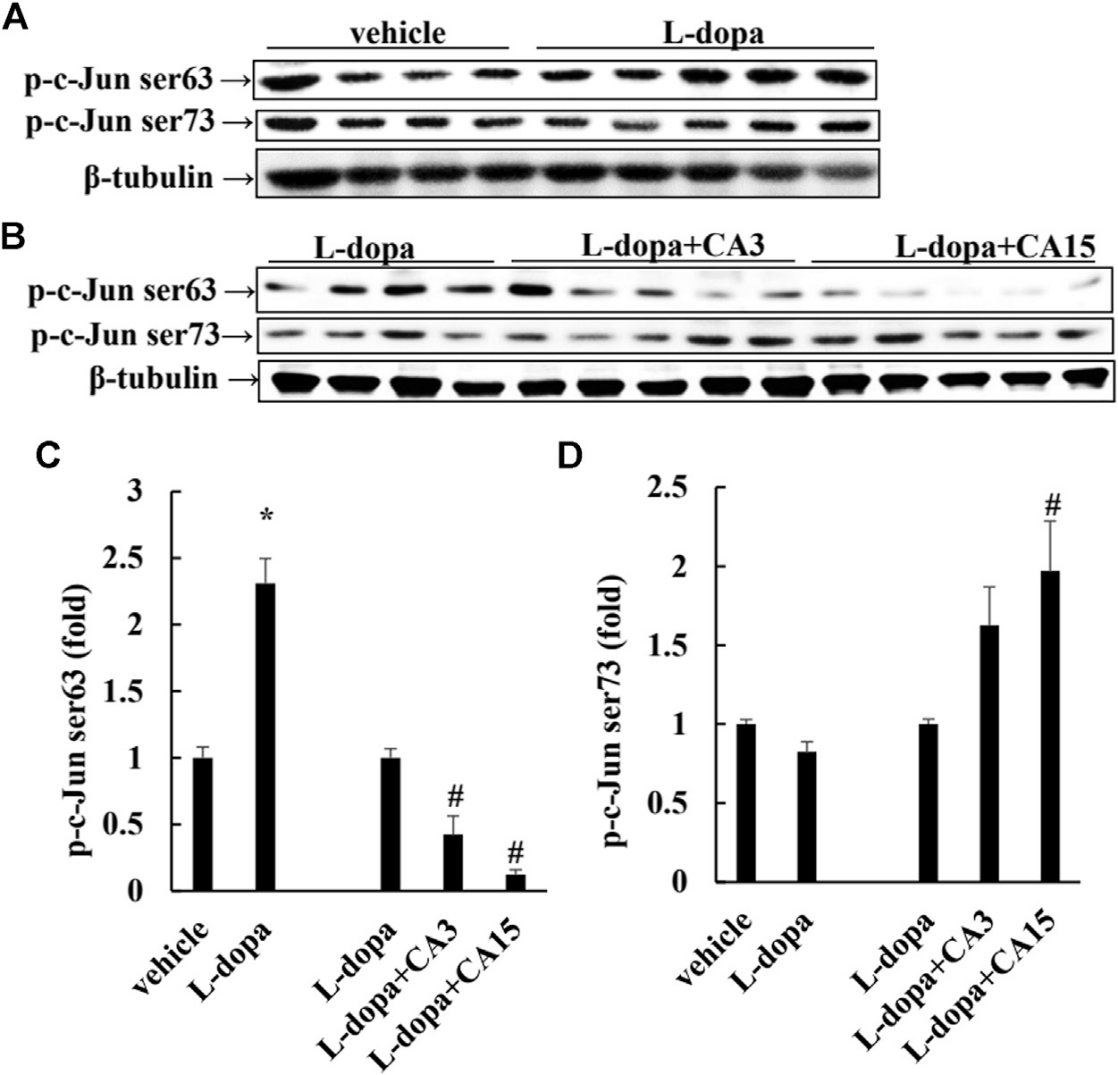
Effect of CA on _L_-dopa-induced activation of ERK1/2 and c-Jun in 6-OHDA lesioned rats. The phosphorylation of c-Jun Ser63 and c-Jun Ser73 in striatum tissues were determined by Western blotting **(A,B)**. β-Tubulin was used as the internal control. **(C,D)** The level of the _L_-dopa group was set at 1.0. Values are mean ± SEM (*n* = 3). #*p* < 0.05 compared with _L_-dopa-treated group.

It has been reported that _L_-dopa induces continuous activation of ERK1/2 to promote c-Jun phosphorylation at Ser63 (as a pro-apoptotic factor) and reduces c-Jun phosphorylation at Ser73 (as an anti-apoptotic factor) (Park et al., 2016). We found that rats treated with _L_-dopa significantly increased the activation of ERK1/2 and c-Jun Ser63 (*p* < 0.05). (Figures 3A, 4A). However, phosphorylation of ERK1/2 and c-Jun Ser63 was reduced in cells pretreated with 3 and 15 mg/kg CA (Figures 3B, 4B). CA at 15 mg/kg reduced the activation of ERK1/2 and c-Jun Ser63 by 60 and 88%, respectively, compared with that in the _L_-dopa group (*p* < 0.05). However, only the group pretreated with 15 mg/kg CA showed an increase in the phosphorylation of c-Jun Ser73 of about 97% compared with the _L_-dopa group (*p* < 0.05) (Figure 4B).

### CA Prevents Apoptosis Induced by _L_-Dopa in SH-SY5Y Cells

**FIGURE 5:**
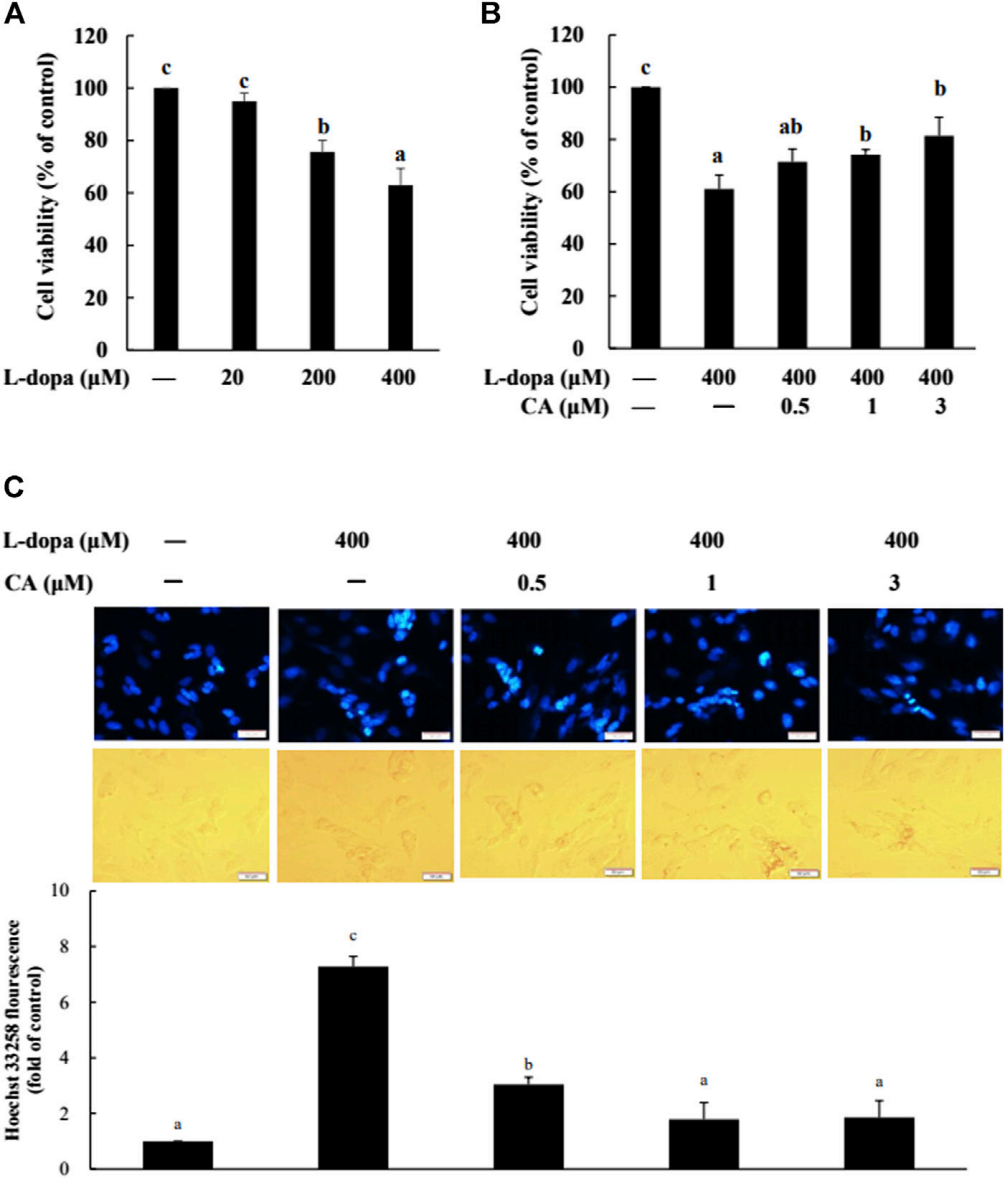
Effect of CA on _L_-dopa-induced toxicity in SH-SY5Y cells. Cell viability was measured by MTT assay. **(A)** Cells were pretreated with 20, 200, and 400 μM _L_-dopa for 72 h. Control (−) was treated with PBS. **(B)** Cells were pretreated with 0.1% DMSO alone (−) or 0.5, 1, and 3 μM CA for 18 h and were then treated with 400 μM _L_-dopa for an additional 72 h. **(C)** Nuclei were visualized with Hoechst 33258 staining. Cells were pretreated with DMSO alone (−) or 0.5, 1, or 3 μM CA for 18 h and were then treated with 400 μM _L_-dopa for an additional 24 h. One representative image out of three independent experiments is shown. Values are mean ± SD (*n* = 3). Means not sharing a common letter are significantly different, *p* < 0.05.

**FIGURE 6:**
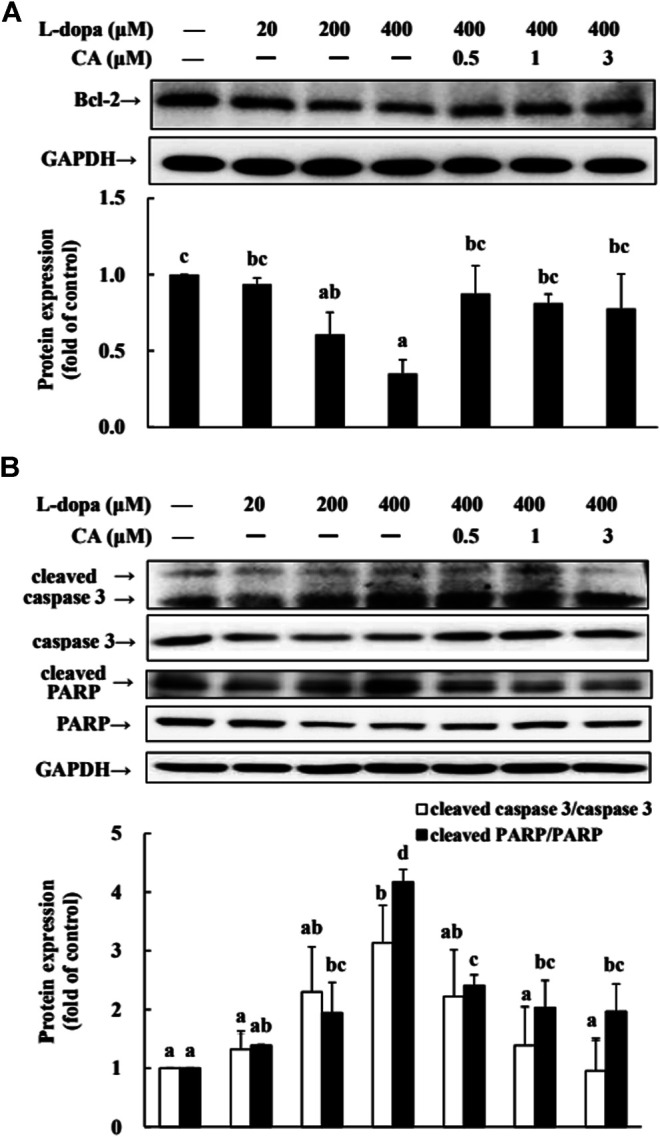
Involvement of reduction in apoptosis-related proteins in the neuroprotective effects of CA. **(A) **Cells were pretreated with DMSO alone (−) or 0.5, 1, or 3 μM CA for 18 h and were then treated with 20, 200, and 400 μM L-dopa for an additional 24 h. One representative immunoblot out of three independent experiments is shown. **(B)** The control group was regarded as 1. Values are mean ± SD (*n* = 3). Means not sharing a common letter are significantly different, *p* < 0.05.

We further used SH-SY5Y cells to explore the effect of CA on cytotoxicity induced by _L_-dopa. The results indicated that _L_-dopa dose-dependently reduced cell viability. Cell viability in cells treated with 400 μM _L_-dopa was reduced by about 36% compared with the control group (Figure 5A) Pretreatment with 1 and 3 μM CA, however, was able to increase cell viability by 21 and 34%, respectively, in _L_-dopa-treated cells compared with that in cells treated with _L_-dopa alone (*p* < 0.05) (Figure 5B). The results of Hoechst 33258 staining were similar, as shown in Figure 5C. Exposure to _L_-dopa induced the intensity of Hoechst 33258 fluorescence, suggesting that _L_-dopa treatment increased nuclear condensation and then induced apoptosis. However, the effect of _L_-dopa on nuclear condensation was reduced in cells pretreated with CA. We then used immunoblotting to examine the effect on proteins related to apoptosis. _L_-Dopa dose-dependently reduced the expression of Bcl-2 protein and increased the ratio of cleaved caspase 3/caspase 3 and cleaved PARP/PARP (Figures 6A,B). CA pretreatment reversed these findings.

### CA Regulates ERK1/2/c-Jun Signaling Induced by _L_-Dopa in SH-SY5Y Cells

**FIGURE 7:**
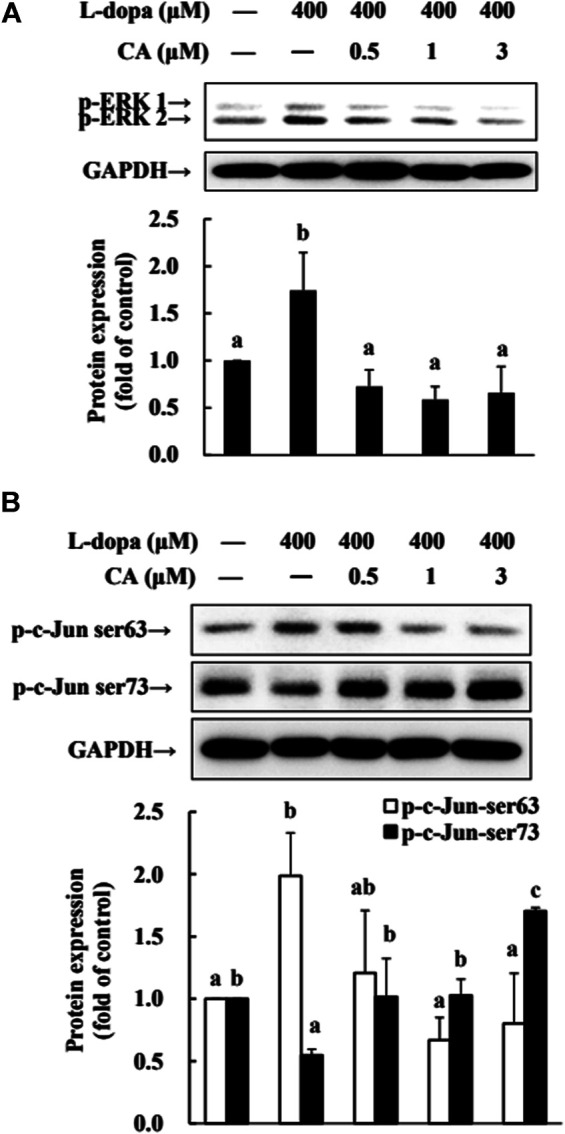
Effect of CA on the activation of ERK1/2 and c-Jun in L-dopa-treated SH-SY5Y cells. **(A)** Cells were pretreated with DMSO alone (−) or 0.5, 1, or 3 μM CA for 18 h and were then treated with 400 μM L-dopa for an additional 0.5 h. One representative immunoblot out of three independent experiments is shown. **(B)** The control group was regarded as 1. Values are mean ± SD (*n* = 3). Means not sharing a common letter are significantly different, *p* < 0.05.

The phosphorylation of ERK1/2 and c-Jun Ser63 was increased in cells treated with _L_-dopa, and the activation of c-Jun Ser73 was reduced (Figure 7). However, pretreatment with CA improved the effects of _L_-dopa on these proteins (*p* < 0.05).

### CA Improves the Expression of Parkin and Ubiquitinated Protein Induced by _L_-Dopa in SH-SY5Y Cells

**FIGURE 8:**
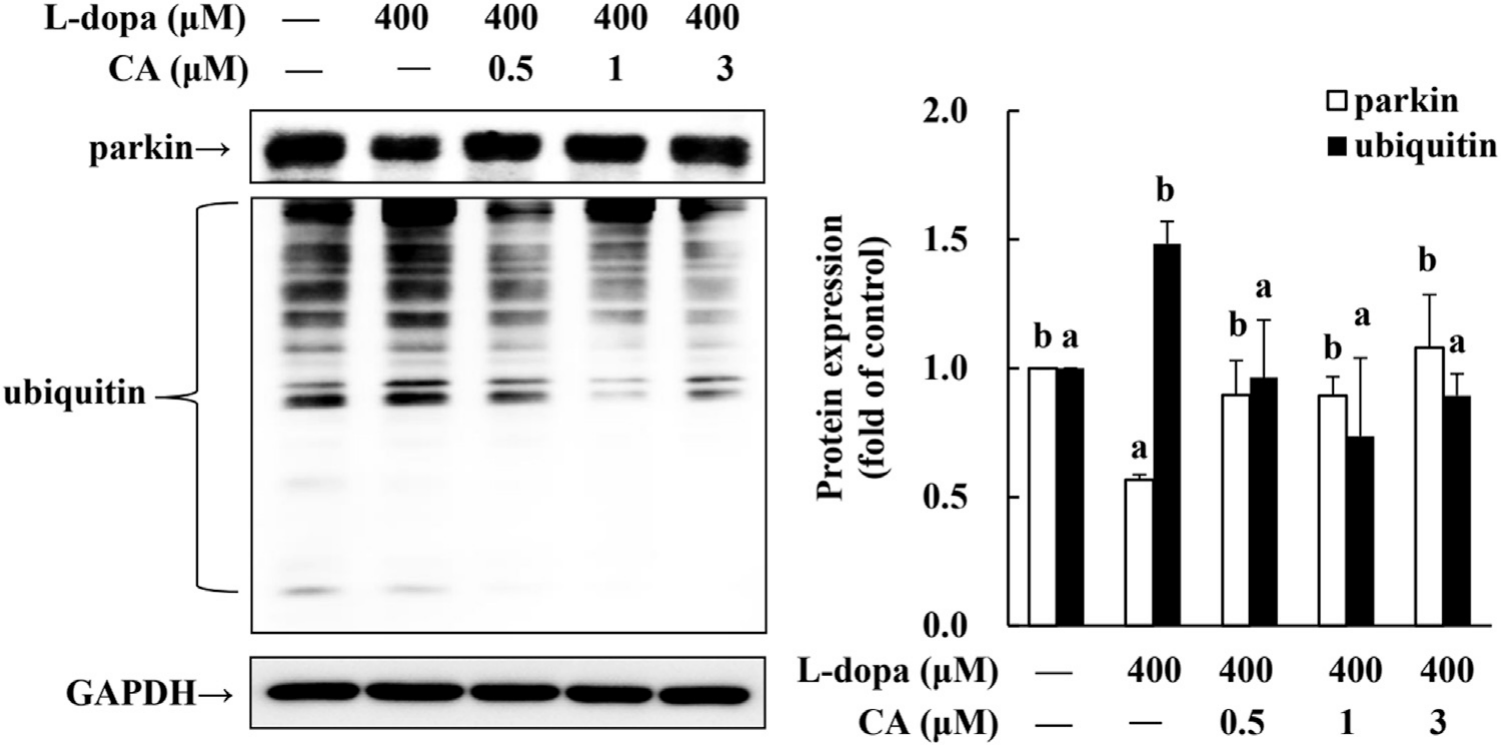
Effect of CA on the expression of parkin and ubiquitinated protein in _L_-dopa-treated SH-SY5Y cells. Cells were pretreated with DMSO alone (−) or 0.5, 1, or 3 μM CA for 18 h and were then treated with 400 μM L-dopa for an additional 24 h. One representative immunoblot out of three independent experiments is shown. The control group was regarded as 1. Values are mean ± SD (*n* = 3). Means not sharing a common letter are significantly different, *p* < 0.05.

Parkin is a ubiquitin protein ligase E3 and plays a critical role in the ubiquitin-proteasome system (UPS) (Higashi et al., 2004). The activation of D1R by _L_-dopa is associated with the dysregulation of the (Berthet et al., 2012). Therefore, in this study, we also examined ubiquitinated-related proteins. We found that parkin protein was reduced and ubiquitinated protein was increased in cells treated with _L_-dopa (Figure 8). CA pretreatment improved both proteins in cells treated with _L_-dopa (*p* < 0.05).

## Discussion

LID is the adverse events after long-term use of _L_-dopa in PD patients (Chaudhuri et al., 2019). Several strategies for overcoming LID have been developed, but some of these have limitations (Ryu et al., 2018). In the current study, we revealed that CA could improve the development of LID in a 6-OHDA-lesioned rat model. CA inhibited D1R-induced signaling, including p-DARPP-32, p-ERK1/2, and ΔFosB. Moreover, we revealed that CA attenuated the levels of cleaved-caspase 3 and -PARP by _L_-dopa is associated with the regulation of the ERK1/2-c-Jun pathway. In addition, we found that CA increased parkin protein to reduce the ubiquitinated protein by _L_-dopa. It is the first study to show the favorable effect of CA against the side effects inducing by _L_-dopa treatment for PD.

LID is a serious motor complication that develops after long-term _L_-dopa therapy for PD (Sun et al., 2020). Study indicated that the time-course of _L_-dopa during 0–60 min exhibits higher total AIM and ALO scores (Ryu et al., 2018). Consistent with these results, our results revealed that the ALO scores are higher after _L_-dopa administration during 0–60 min. CA group decreased the ALO scores during 20–60 min. Moreover, CA treatment exhibited lower the ALO scores up to 33 days. These results suggested that CA is beneficial for long-term treatment of LID for PD. Recent studies have reported that the behavioral expression of LID is associated with D1R signaling. However, the signaling was unchanged by continuous administration of _L_-dopa through a subcutaneous mini-pump, suggesting prevention the fluctuation of _L_-dopa concentrations relieved the LID (Lebel et al., 2010). In the present study, CA attenuated the activation of D1R/DARPP32/ERK1/2 cascade and subsequently led to counteract the effect of LID in 6-OHDA-lesioned rats. Because D1R signaling is triggered by the activation of PKA, study reported that treatment with PKA inhibitor Rp-cAMPS alleviated the LID in 6-OHDA-lesioned rats (Lebel et al., 2010). Similarly, researchers showed that blocking ERK1/2 phosphorylation by SL327 reduces LID in 6-OHDA-lesioned mice treated with _L_-dopa (Santini et al., 2007). Study also indicated that ΔFosB is accumulated after abusing some drugs and increased the sensitivity to the behavioral effects (Nestler et al., 2001). The present study found that striatal ΔFosB protein induction is related with the AIM and ALO scores by _L_-dopa. In parallel with behavior reversal, CA treatment reduced D1R/DARPP32/ERK1/2 cascade and decreased ΔFosB protein caused by _L_-dopa treatment.

Research shows that ERK1/2 activation is involved in the neuronal cell viability induced by _L_-dopa treatment (Park et al., 2016). Treatment of PC12 cells with _L_-dopa induces the activation of ERK1/2 and caspase 3 (Jin et al., 2010; Park et al., 2014). Similarly, in 6-OHDA-lesioned rats, administration of _L_-dopa upregulated the ERK1/2 activation, leading to increase c-Jun phosphorylation at ser63, but decrease c-Jun phosphorylation at ser73 (Park et al., 2016). It is because _L_-dopa at high concentrations was cytotoxic and stimulated the activities of ERK1/2 and caspase 3. These results could explain that PD patients after long-term exposure to _L_-dopa increased neurotoxic events (Blandini et al., 2004) and stimulated abnormal involuntary movements (Chaudhuri et al., 2019). Our results in the present study supported the report that _L_-dopa increased ERK1/2/c-Jun activation and enhanced caspase 3 activation (Figures 3, 4, 6, 7). CA treatment could increase the cell survival through reducing the ERK1/2 activation and alleviating the alternation of c-Jun pathway to prevent apoptotic neuronal cell death.

Recently, evidence reported that dysregulation membrane localization of dopamine D1R impairs the striatal ubiquitin-proteasome system and increases the accumulation of ubiquitinated protein to exaggerate the D1R transmission (Barroso-Chinea et al., 2015). Parkin, an ubiquitin E3 ligase, facilitates multiple misfolded proteins degradation by 26S proteasome, and plays an important role in neuroprotection (Wilhelmus et al., 2012). Study reported that knockdown of parkin displays the higher AIMs scores than in wild-type mice. Moreover, compare to control mice, parkin mutant mice shows an earlier onset of AIMs (Berthet et al., 2012). Our previous study indicated that CA acts to attenuate 6-OHDA-induced neurotoxicity associated with the induction of parkin through enhancing UPS and preventing apoptosis (Lin et al., 2016). In this study, we have shown that SH-SY5Y cells treated with _L_-dopa decreased the parkin and increased the ubiquitinated protein; however, CA pretreatment reversed parkin and ubiquitinated proteins by _L_-dopa. Therefore, we speculated that CA up-regulated the parkin protein and reduced the D1R abnormal trafficking, leading to alleviation of D1R signaling and LID.

**FIGURE 9:**
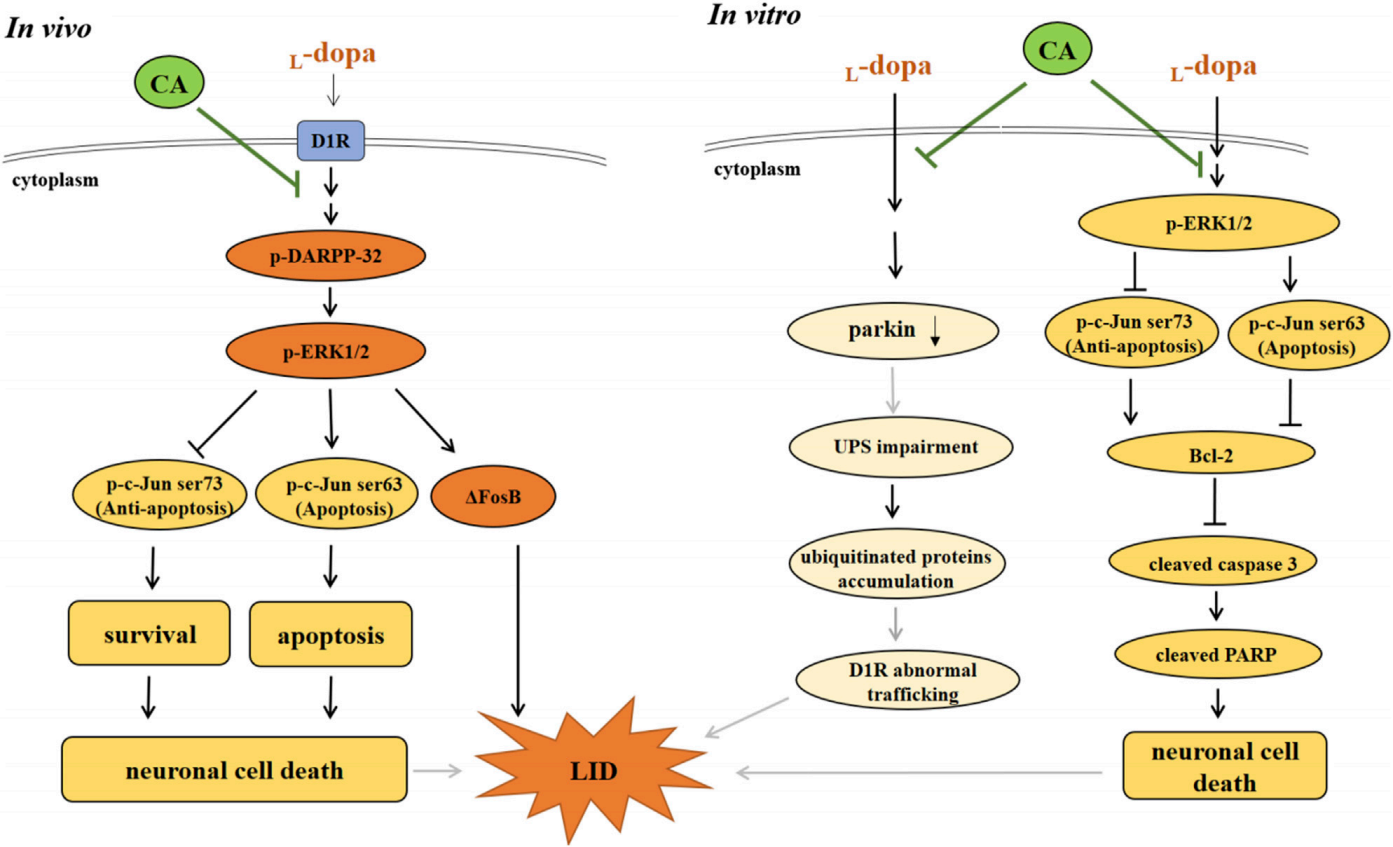
The actions of neuroprotective mechanism of CA on LID in *in vivo* and *in vitro* studies. **(A)** In *in vivo* study, _L_-dopa stimulates DIR protein, and then activates the phosphorylation of DARPP-32 and ERK1/2, which elevates ΔFosB protein, leading to develop the LID in 6-OHDA-lesioned rats. Moreover, _L_-dopa administration induced neuronal cell death through regulating ERK1/2-c-Jun pathway. However, CA alleviates these effects induced by _L_-dopa. **(B)** In *in vitro* study, pretreatment of CA with SH-SY5Y cells attenuates _L_-dopa-triggered apoptotic cell death mediated *via* increasing c-Jun ser73 activation and decreasing c-Jun ser63 activation by ERK1/2. Additionally, CA could be improved the development of LID is related to the induction of parkin protein, leading to prevent the ubiquitinated protein accumulation and D1R abnormal trafficking (gray line).

In conclusion, the results of the current indicate that CA can alleviate LID-induced behavior changes in 6-OHDA-treated rats by regulating the D1R-mediated activation of DARPP-32 and ΔFosB. The protective mechanisms of CA are involved with the inactivation of ERK1/2/c-Jun pathway and the induction of parkin protein, leading to reduction of apoptotic neuronal cell death and LID (Figure 9). CA could be recommended as a beneficial therapy for delaying the development of LID in PD patients.

## Data Availability

The raw data supporting the conclusion of this article will be made available by the authors, without undue reservation.
